# Health Tract, No. 249

**Published:** 1866-06

**Authors:** 


HEALTH TRACT, No. 249.

### MEDICINAL TERMS.

*Anodynes*, cause sleep, as opium, hops &c.

*Astringents*, bind, close up, contract, as vinegar and the persimmon.

*Cathartics* empty the bowels by purging, as salts and castor oil.

*Diaphoretics* cause perspiration, as hot herb tea.

*Emetics* empty the stomach, as tartar, Ipecac, tobacco, &c.

*Expectorants* loosen the phlegm in the lungs.

*Irritants* draw the blood to the part away from the painful spot, and thus relieve, as a mustard plaster; thus giving the ailing part time to heal.

*Liniments* are irritant, in a liquid form.

*Lotions* are washes to cleanse or soothe.

*Refrigerants* are to cool in fevers, as acids, lemonades, &c.

*Tonics* are intended to give strength, as bitters, made of vegetable remedies.

The practice of medicine consists in knowing what is the matter, what is needed, and what will accomplish the object. The first requires observation, the second judgment, the third experience, and he who possesses these in the greatest measure will always be the most successful physician, however great may be the intelligence or ignorance in other directions. The physician who is master of his profession, knows what part of the body is affected, how it is affected and what will remove the affection, all that is uncertain is, "Will the ship answer to the helm?" Will the constitution in a specific case, be capable of being acted on by a remedy and have the power to rise, after such action? If a mustard plaster is applied in an external case to the ankles, and there is life enough for it to draw, the man is saved; if not, all the vitality is gone and he dies.

A physician learns by appearances or feelings what is the matter with a man, what part of the body is affected, and knowing what medicine usually acts on that part, he gives it, and the man is saved. He cannot tell why a certain remedy affects a certain part, but he knows that it does and that is sufficient. Spirits "act on" the brain; fumes on the lungs; ipecac, on the stomach; rhubarb on the upper bowels; aloes on the lower; mercury on the liver; watermelons on the kidneys; strychnyne, on the nerves; ergot, on the womb.

Brandy makes a man as funny as a fool. Opium makes him as stupid as an ass. A hop infusion will put him to sleep. Tea keeps him wide awake. It is on these facts, and principles, that the whole science of medicine is founded; principles on solid as the Cordilleras, and as lasting as the ages; hence, those whose prejudices prevent them from taking medicine in case of sickness are constructive suicides, and he who derides the healing power of physic is a fool.

